# A genomic locus uniquely encoded by blueberry-infecting *Xylella fastidiosa* strains affects motility and biofilm formation in vitro, and virulence in planta

**DOI:** 10.1371/journal.pone.0346230

**Published:** 2026-04-03

**Authors:** Navdeep Kaur, Marcus V. Merfa, Alexandra K. Kahn, Rodrigo P. P. Almeida, Leonardo De La Fuente

**Affiliations:** 1 Department of Entomology and Plant Pathology, Auburn University, Alabama, United States of America; 2 Department of Microbiology, University of Tennessee Knoxville, Knoxville, Tennessee, United States of America; 3 Department of Environmental Science, Policy, and Management, University of California, Berkeley, California, United States of America; University of Salento Department of Biological and Environmental Sciences and Technologies: Universita del Salento Dipartimento di Scienze e Tecnologie Biologiche ed Ambientali, ITALY

## Abstract

*Xylella fastidiosa* (*Xf*) is an insect-transmitted, xylem-limited bacterial plant pathogen that infects hundreds of plant species. This pathogen causes bacterial leaf scorch in southern highbush blueberry (*Vaccinium corymbosum* interspecific hybrids) in the southeastern United States, a disease that has not yet been reported elsewhere. Previously, a comparative genomic analysis of *Xf* and ancestral host species identified evolutionary events of gene gain and loss related to host range specificity. Here, by using a similar workflow, we identified two loci that are significantly found in blueberry-infecting strains. Locus_1088 included a hypothetical protein and a small part of the N-terminus of an orphan RelE toxin, while Locus_2741 was annotated as a hypothetical protein. Using a protocol based on natural competence, mutants were generated in three *Xf* subsp. *multiplex* strains from blueberry. Less biofilm, more planktonic growth, and increased twitching motility as compared to its wild-type (WT) were observed for the strain LA-Y3C_1088 mutant. In blueberry virulence assays, the LA-Y3C_1088 mutant caused significantly more severe symptoms than LA-Y3C_WT, whereas no significant differences were observed for other mutated strains. Interestingly the mutation of Locus_1088 additionally disrupted a toxin (part of a toxin-antitoxin system) that is likely responsible for the phenotypic changes observed. However, because the two independent mutants were not generated, we could not determine whether the phenotype resulted from disruption of hypothetical protein or the toxin. Additionally, since the coffee-isolated but never tested in blueberry *Xf* subsp. *fastidiosa* strain CFBP8073 was found to encode the two blueberry-associated loci studied here, its virulence was assessed in blueberry. This strain caused severe symptoms comparable to the control strain AlmaEm3 from blueberry. Due to the complexity of understanding host specificity in *Xf,* any advance in identifying genetic markers for host specificity in this devastating pathogen could greatly improve management of *Xf* worldwide.

## Introduction

*Xylella fastidiosa* (*Xf*) is a slow-growing bacterium that inhabits the xylem vessels of its plant hosts and is transmitted by xylem sap-feeding insects [1–4]. This γ-proteobacterium is known to cause diseases in hundreds of plants such as grapevine, citrus, olive, peach, almond, blueberry, and plum, and it is also found as an endophyte in many plant species [5]. *Xf* has been primarily classified into three subspecies: *Xf* subsp*. fastidiosa*, *multiplex* and *pauca* [6]. In the past, it was believed that different subspecies had almost non-overlapping host ranges [7]. But recent studies have suggested that strains from different subspecies have broader host ranges than previously thought [8–10].

Host specificity in plant-associated bacteria has primarily been studied in relation to the production of specific effectors secreted via the type III secretion system, which then interact with plant proteins [11]. However, *Xf* does not have a type III secretion system and therefore lacks effectors that can be secreted with this system [12]. Several studies have indicated a possible host-specific relationship between *Xf* and plant species [13–15], and additional host specialization has been reported within subspecies [16,17]. Therefore, identifying factors influencing host specificity remains essential for understanding the distribution and impact of this pathogen.

In a previous study [18], the disruption of *rpfF*, which is responsible for synthesis of the quorum-sensing molecule Diffusible Signaling Factor (DSF), affected host range, indicating that gene regulation may be one of the determinants of host specificity in *Xf*. Several studies tried to correlate host specificity with core genome phylogenetic relationships, suggesting that host specificity might be coded in the accessory genome [19]. A recent study has established a link between pseudogenes and host specificity, identifying candidate host-specific sequences through comparative genomics analysis of different *Xf* strains [20]. Using comparative genomics and phylogenetics methods, another study demonstrated that *Xf* strains host-specificity is correlated with their ancestral plant hosts, but not necessarily the host species of isolation, indicating that there is a genetic basis for the host range of *Xf* [6]*.* This study identified a group of candidate genes associated with hosts and was used as the basis for the present study to experimentally assess the function of some of these loci.

Our study tested the hypothesis that two loci, identified using a reverse genomics approach [6], are important for colonization of blueberry by *Xf* and subsequent disease development. We focused on this crop because highbush and rabbiteye blueberry (*Vaccinium corymbosum* interspecific hybrids and *V. ashei*, respectively) are a valuable fruit crop in the southeastern US region; for instance, the state of Georgia is the fourth-largest producer in the US [21]. Mutation of one of these loci in one of the three tested strains used here caused pronounced changes *in vitro* motility and growth, and more severe symptoms in blueberry under controlled conditions. Moreover, based on the loci studied here, we identified a previously intercepted *Xf* strain isolated from coffee, that was able to cause severe symptoms in blueberry. Here, we provide experimental evidence of the usefulness of this approach of identifying candidate host-specific loci via comparative genomics. Although we are aware that our methodology is limited and is unable to determine which of the two genes (hypothetical protein or toxin) in Locus_1088 may be responsible for the phenotype observed, this study nonetheless represents a valuable contribution towards understanding the basis of virulence in this difficult pathogen.

## Materials and methods

### Selection of loci sequences for deletion

A previous study identified a group of candidate *Xf* genes associated with specific host families based on comparative genomics and phylogenetic analysis of isolates [6]. For this study, a similar comparative genomics analysis was carried out including additional *Xf* isolates from blueberry. Briefly, core and pan genomes were built using Panaroo [22] on 335 whole genome annotations from all subspecies of *Xf* (annotated using Prokka [23], including 4 subsp. *multiplex* strains isolated from *Vaccinium* spp., the genus to which blueberry belongs (strain metadata can be found in S1 Table). A matrix was built of the pan genome gene presence and absence and run through Scoary [24] to determine correlation between the isolation host and gene presence or absence (S2 Table) using Fisher’s Exact Test with the Benjamini-Hochberg correction with a significance threshold set at p ≤ 0.05 [25].

### Visualization of Locus_1088 genomic neighborhood

The genomic neighborhood of Locus_1088 in strains LA-Y3C (GenBank accession number CP090510.1), AlmaEm3 (GenBank accession number CP072933.1), and BB08−1 (GenBank accession number CP072932.1) was obtained by uploading genomes to the Benchling cloud-based platform (<www.benchling.com>) to extract the sequences of genomic neighborhoods in.gbk format. Then, these sequences were submitted to cluster comparison analysis via Clinker v0.0.23 [27] (default settings) to generate Fig 2.

**Fig 1:**
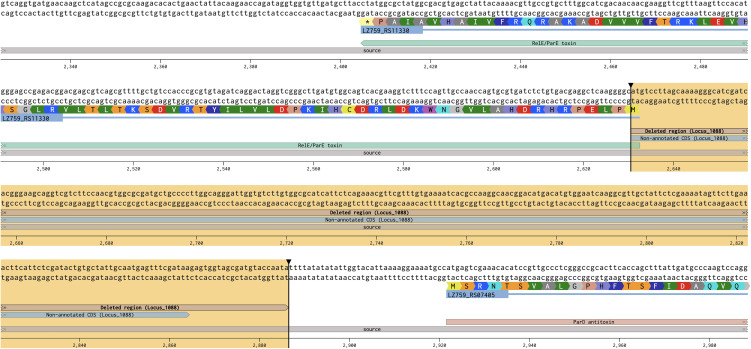
Deletion of Locus_1088 in blueberry *X. fastidiosa* (*Xf*) strains. The deleted genomic region for Locus_1088 in strain LA-Y3C is highlighted in the figure as a representation of this deleted genomic region in blueberry *Xf* subsp. *multiplex* strains. The non-annotated CDS (light blue annotation) for Locus_1088 is also included in the figure inside the highlighted deleted region. The figure was generated by downloading the genome (.gbk; GenBank accession number CP090510.1) of strains LA-Y3C from NCBI and visualizing the genomic region via Benchling (<www.benchling.com>).

### Bacterial strains and culturing conditions

All strains were retrieved from frozen glycerol stocks stored at −80°C and streaked onto Periwinkle Wilt (PW) [28] agar plates, followed by incubation for one week at 28°C. Afterwards, these cultures were re-streaked onto fresh PW agar plates and incubated for an additional week. Antibiotic resistance selective PW agar plates were prepared by adding Kanamycin (Km) at a concentration of 30 µg/ml for the strains in which the identified loci were deleted. For cell suspension and culturing, Pierce’s Disease 2 (PD2) [29] and PD3 [30] media were used.

### DNA Extraction and knockout template construction

Cells from wild type (WT) *Xf* strains cultured on PW agar plates were suspended in 200 μL of sterile Milli-Q water (Merck Millipore, Burlington, MA) and stored at −20°C until needed. DNA was extracted using a modified cetyltrimethylammonium bromide (CTAB) extraction protocol [31]. Deletion of loci was performed following a previously published protocol [32]. Mutants were named using the background strain name and the knockout locus sequences as follows: AlmaEm3_1088, LA-Y3C_1088, BB08–1_1088, and BB08–1_2741. Briefly, to amplify the upstream and downstream regions of the chosen loci from all three genomes, 800 bp from both upstream and downstream regions were used. Amplification was performed with the respective UP_F/UP_R and Dn_F/Dn_R primers (S4 Table).

**Fig 2:**
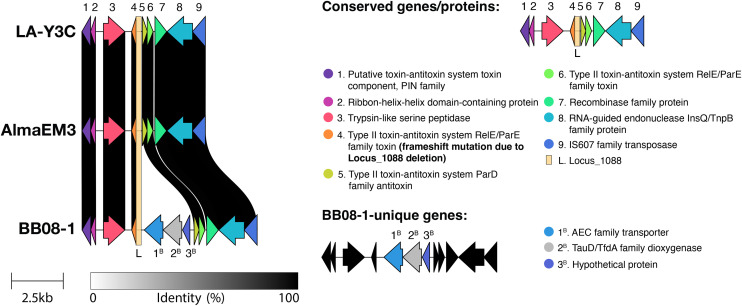
Schematic overview of the Locus_1088 neighborhood types in blueberry-infecting *X. fastidiosa* strains obtained using Clinker. There is a toxin-antitoxin (TA) system (ParD family antitoxin and RelE/ParE family toxin; genes #5 and #6 in the figure, respectively) directly downstream to the Locus_1088 and the toxin gene (RelE/ParE family toxin; gene #4 in the figure) that was targeted for a frameshift mutation in this study. The locus neighborhood type is similar for all strains. However, strain BB08−1 has an insertion of three genes between the deleted toxin gene and the TA system downstream to it. L – Locus_1088 that is highlighted in the figure. 1^B^, 2^B^ and 3^B^ – unique genes found in strain BB08−1 in its Locus_1088 neighborhood type.

The upstream and downstream regions of AlmaEm3_1088 and LA-Y3C_1088 were 99.38% and 100% identical, respectively, leading to the design of a single pair of primers for their amplification. In contrast, the upstream and downstream regions of the target locus sequence for BB08–1 were not identical to those of AlmaEm3_1088 and LA-Y3C_1088; therefore, a different set of primers was designed for amplifying these regions (S4 Table). Curiously, the identified Locus_1088 includes a coding sequence (CDS) that is not annotated in blueberry-infecting *Xf* genomes obtained from NCBI (Fig 1). No functional domain was identified when the translated sequence of this CDS (obtained via Expasy Translate tool; < web.expasy.org/translate/>) was searched using the NCBI Conserved Domains tool (<www.ncbi.nlm.nih.gov/Structure/cdd/wrpsb.cgi>). Nonetheless, deletion of Locus_1088 also deleted the first two nucleotides of an annotated CDS encoding a RelE/ParE toxin (Fig 1). This caused a frameshift mutation in the toxin gene that likely led to a non-functional CDS, which was observed by aligning the original RelE amino acid sequence with the frame-shifted amino acid sequence (no significant similarity was found). The gene encoding this orphan ortholog of the RelE/ParE toxin is also unique to blueberry-infecting *Xf* strains, which was verified via blastx. While *X. fastidiosa* genomes regularly encode a *relE* toxin gene (ranging around ~99% coverage and ~88–99% similarity) alongside the cognate gene encoding the antitoxin, only blueberry-infecting strains encode the orphan *relE* toxin gene. The orphan RelE and regularly encoded RelE toxins share ~62–67% coverage and 94–98% similarity. Additionally, the genomic neighborhood of Locus_1088 includes a type II toxin-antitoxin (TA) system composed of a ParD antitoxin and another copy of a RelE/ParE toxin directly downstream to Locus_1088 (Fig 2).

Primers were also designed for Locus_2741 as follows: upstream regions of AlmaEm3_2741 and BB08–1_2741 were 100% identical and the same primers were used to obtain the upstream constructs. AlmaEm3_2741 is duplicated in the genome (two identical copies), and there were complications in knocking out the target region, and we were unable to obtain a mutant. Accordingly, a mutant was obtained only for strain BB08_2741. In contrast to Locus_1088, Locus_2741 corresponds to a CDS that is annotated as a hypothetical protein in the blueberry-infecting *Xf* genomes available on NCBI (S5 Table). These two loci were targeted for knockout via homologous recombination with a Km-resistance cassette [32]. The cassette encoding Km resistance was amplified from plasmid pUC4K [33] with a previously designed primer pair [32].

DNA from all three WT strains, i.e., LA-Y3C, AlmaEm3, and BB08–1 was used as a template to amplify their respective upstream and downstream regions of the locus of interest [33]. Primers Up_R and Dn_F were modified at their 5’ ends to include at least 21 bp of homology to the Km resistance cassette, allowing the joining of the upstream and downstream genomic fragments to the antibiotic resistance cassettes using overlap extension PCR (initial denaturation at 98°C for 2 minutes, 35 cycles of denaturation (98°C for 20 seconds), annealing (59°C for 30 seconds), extension (72°C for 2 minutes), and final extension (72°C for 5 minutes). For this, the three individually amplified fragments, i.e., upstream, antibiotic cassette, and downstream were purified from the agarose gel, and mixed in equal proportions for an overlap extension PCR using the end primers (UP_F and Dn_R). The resulting fusion product, containing the antibiotic resistance cassette flanked by genomic sequences upstream and downstream of the target, was purified from the agarose gel and stored at −20°C for future use.

All primers were designed manually, and their quality was assessed using PCR Primer Stats tool (<bioinformatics.org>). Following the design of the primer sets, the Primer–BLAST tool on NCBI was used to verify primer specificity against the target genome, ensuring that each pair annealed to only a single region. PCR was performed with a 2x iProof high fidelity master mix (Bio-Rad), in an S1000 thermal cycler (Bio-Rad, Hercules, CA). PCR products were gel-purified using IBI Scientific Gel/PCR DNA Fragments Extraction kit.

### Loci deletion using natural transformation

A natural transformation-based protocol was used to mutate *Xf* cells using the gel-purified PCR templates described above [32], with the goal of inserting a Km resistance cassette and disrupting the target loci. Briefly, recipient strains AlmaEm3, LA-Y3C, and BB08−1 strains were cultured on PW media for 7 days, then re-streaked and incubated for another 7 days. Strains were subsequently suspended in PD3 liquid media, and the optical density (OD_600_) adjusted to 0.25. The suspension (10 µL) was placed on PD3 agar plates, followed by the addition of 10 µL of knockout templates (see above) on top of the same spot. Control spots consisting of bacteria where buffer was added instead of DNA were included in each experiment. The spots were allowed to dry before the plates were incubated at 28°C for 3 days. After incubation, the spots were suspended in 500 μL of PD3, and 100 μL of aliquots were spread plated onto PW plates containing Km (30 μg/ml) for selection. After two weeks, mutant and WT colony forming units (CFUs) were re-streaked onto new antibiotic plates. Five colonies per strain per construct were selected and re-streaked onto new antibiotic plates. Confirmation of loci deletion and recombination in the genome was performed via PCR using Taq 5x master mix (New England Biolabs, Ipswich, MA) [32] (S4 Table).

### In-vitro mutant characterization

All experiments included in this section were independently repeated three times, except for the settling rate measurements that were repeated twice.

#### i. Growth curves and biofilm formation.

Growth curves and biofilm formation of both mutant and WT strains were evaluated in 96-well plates over a period of 7 days, as previously described [34]. Each experiment was conducted independently three times, using three separate 96-well plates for each trial in PD3 media. Sterile polystyrene 96-well plates were filled with 190 μL of PD3 per well and inoculated with 10 μL of 0.2 OD_600_ bacterial cell suspension to make final OD_600_ of 0.01 in each well (or PD3 media in control wells). The OD_600_ of samples was measured daily to obtain growth curves of assayed strains. After incubating the plates for 7 days at 28°C, planktonic growth was measured by transferring the cell-containing broth to a new plate. To evaluate biofilm formation, the original plate was rinsed three times with Milli-Q water and stained with 0.1% crystal violet [35]. On each plate, there were 10 replicates for both the wild type and mutant strains.

#### ii. Twitching motility.

The twitching motility of both wild type and mutants was assessed on PW plates without BSA as previously described [36]. The solidified agar media plates were divided into two halves, with 10-12 colonies from each strain spotted on each half. The plates were incubated at 28°C for 5 days [34]. Colony peripheral fringes were examined at 10x magnification using a Nikon Eclipse Ti inverted microscope (Nikon, Tokyo, Japan), and fringe widths were measured for six colonies from two plates per strain, taking four measurements for each colony. The experiment was independently repeated three times.

#### iii. Settling rate.

Cell-to-cell aggregation was assessed using the settling rate method as previously described [35] with a UV-Vis 2450 spectrophotometer (Shimadzu Scientific Instruments, Columbia, MD). Briefly, cell suspensions were thoroughly mixed by pipetting and then placed into the spectrophotometer, where OD600 was continuously recorded for 10 minutes. The settling rate was determined by calculating the slope of the linear segment of the decrease in OD600 over time [35].

### Virulence assessment in the greenhouse

Southern highbush blueberry plantlets (*Vaccinium corymbosum* interspecific hybrids, cultivar Rebel), were purchased from Agri-Starts (Apopka, Florida), and acclimatized in the greenhouse for one week [37]. A soil mix of pine bark and sand in a 3:1 ratio was prepared, with the soil pH adjusted to 4–5. The plants were initially transplanted into 4-inch pots, and after approximately one month, moved to 1-gallon pots. During each transplantation, Osmocote Pro (Scotts Company, Marysville, OH) fertilizer was applied; ½ teaspoon during 4-inch pots transplantation, and 1 teaspoon during 1-gallon pots transplantation. All isolates (Table 1) were retrieved from −80°C glycerol stocks and cultured on PW agar plates for seven days at 28°C. These were then sub-cultured onto fresh PW agar plates for an additional seven days at the same temperature. As mentioned above, the mutants were grown on PW agar plates supplemented with Km. Phosphate-buffered saline (PBS, pH = 7) was used to suspend bacterial cells, and an inoculum of OD600 of 0.8 (measured with Thermo Scientific, Genesys 10S Vis Spectrophotometer) was used for inoculating the plants. Plant inoculation was conducted following previously described methods [9]. Briefly, bacterial droplets of 20 μL were applied to the basal region of stems that were wounded by needle pricking, with a second inoculation performed three weeks later. Control plants were inoculated with PBS only. The experiment was performed once. In each treatment group, nine plants were inoculated, with three branches inoculated per plant.

**Table 1:** *Xylella fastidiosa* isolates used in this study.

Strain	Subspecies	Isolation host	WT isolation location, country	References
LA-Y3C_WT	*multiplex*	Blueberry	Louisiana, USA	[26]
LA-Y3C_1088	*multiplex*	Mutant	–	This study
AlmaEm3_WT	*multiplex*	Blueberry	Georgia, USA	[9]
AlmaEm3_1088	*multiplex*	Mutant	–	This study
AlmaEm3_2741	*multiplex*	Mutant	–	This study
BB08–1_WT	*multiplex*	Blueberry	Florida, USA	[9]
BB08–1_1088	*multiplex*	Mutant	–	This study
BB08–1_2741	*multiplex*	Mutant	–	This study
CFBP8073	*fastidiosa*	Coffee	Mexico, intercepted in France	[25]

Blueberry plants were evaluated for the initial appearance of disease symptoms after inoculation (detected ~15 weeks after inoculation), and disease severity ratings were recorded weekly for a period of eight weeks after the first symptoms appeared. The severity of each stem branch on the plant was assessed using a 0-to-7 scale and then averaged for each plant, following the method described by [9]. For calculating disease severity index (DSI%), the following formula was used: (Sum of all individual ratings of stem branches per plant/ No. of plant examined x Maximum disease rating scale) x 100 [10]. After each experiment, the severity scores recorded at nine different time points were used to calculate the area under the disease progress curve (AUDPC) for each inoculated plant. This was done using the midpoint rule method with the formula: AUDPC = Σ[(yi + yi + 1)/2] (ti + 1 − ti), where ‘i’ represents the number of assessment times, ‘y’ is the DSI (%) score for each plant at each assessment, and ‘t’ is the time of each assessment in days [38].

### Quantification of bacterial population

Four to five leaf samples from two positions each (basal and top) from three selected plants were collected to assess *Xf* populations. Petiole samples of around 100 mg were placed into 2 mL tubes containing 2.0 mm zirconia beads, and ground at high speed for three minutes using a Mini-bead beater-96 (Biospec products, Bartlesville, OK). A modified CTAB protocol was used to extract DNA from the samples [31]. The populations of *Xf* were measured using qPCR (TaqMan) with primers HL5/HL6 and a FAM labeled probe [39] along with a standard curve as described previously [40].

### Statistical analysis

All the analyses were performed in *R* version 4.2.2 [41] and *RStudio* [42]. Data were checked for normal distribution using Shapiro-Wilk test, and with the exception of settling rate data, all other data had a non-normal distribution. Groupwise comparisons between *Xf* population means as well as AUDPC means were performed using Kruskal-Wallis test followed by Pairwise Wilcoxon test with *p* < 0.05. For in vitro experiments, such as biofilm formation, planktonic growth, and fringe width measurements, the mean comparisons were statistically tested using Kruskal-Wallis test followed by Pairwise Wilcoxon test with *p* < 0.05. For settling rate, the mean comparisons were statistically tested using one-way ANOVA followed by Tukey’s honestly significant difference method with *p* < 0.05.

## Results

### Loci found to be significantly associated with *Vaccinium* spp.

Locus_1088 was present in 4 of 4 *Xf* subsp. *multiplex* blueberry strains (BB08–1_FL, BBI64_GA, BB01_GA, and AlmaEM3_GA) and only 5 of 331 non-blueberry strains (Sensitivity = 100, Specificity = 98.5) and was thus selected as one locus to knock out. Locus_1088 was significantly associated with host *Vaccinium* spp. (Fisher’s exact test, Benjamini-Hochberg corrected p = 4.03 × 10 ⁻ ⁴). Locus_2471 was also selected since it was present in 3 of 4 blueberry strains and 7 of 331 non-blueberry strains (Sensitivity = 75, Specificity = 97.9). Locus_2471 was significantly associated with host *Vaccinium* spp. (Fisher’s exact test, Benjamini-Hochberg corrected p = 3.78 × 10 ⁻ 3). Full Scoary results can be found in S3 Table. BLASTn was used to compare these two loci sequences against the whole genome database of *Xf*. It was found that Locus_1088 sequence had 100% query coverage and over 90% sequence identity with blueberry-infecting strains LA-Y3C, AlmaEm3, AlmaReb6 and BB08−1. Another genome, *Xf* subsp. *fastidiosa* CFBP8073 (isolated from coffee, *Coffea canephora*) (Jacques et al. 2016), showed 93% query coverage with approximately 89% sequence identity. The *relE* toxin gene upstream of Locus_1088 had 100% query coverage and 100% identity for LA-Y3C, AlmaEm3, and BB08−1 (Fig 1). For Locus_2741, AlmaEm3 (two copies), AlmaReb6, and BB08−1, all exhibited greater than 90% sequence identity with 99% query coverage. The two copies of AlmaEm3 were 100% identical and were separated by a ~ 8kb fragment. Notably, LA-Y3C did not show any hits in the BLAST results for this sequence. Similarly, CFBP8073 showed 100% sequence identity and 100% query coverage. Based on this analysis, in vitro experiments were conducted with LA-Y3C, AlmaEm3, and BB08−1 (Table 1), as representative *Xf* subsp. multiplex strains infection blueberry in the US.

### Deletion of Locus_1088 in strain LA-Y3C decreased biofilm formation in vitro

**Fig 3:**
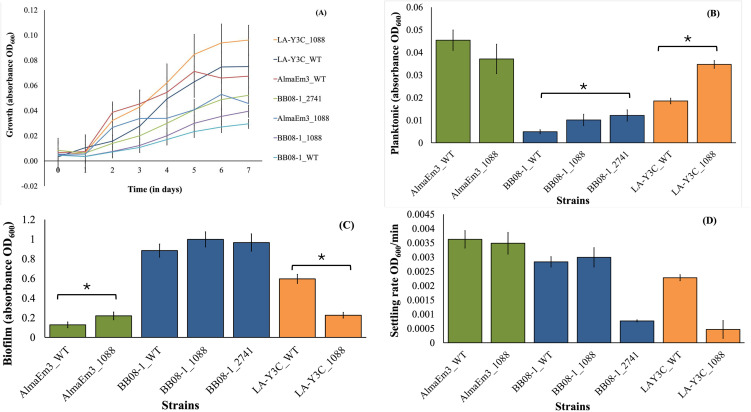
Phenotypic characterization of wild type and mutant strains in vitro. Growth curves (A), planktonic growth (B), biofilm formation (C), and settling rate (D) of *X. fastidiosa* strains. Vertical lines represent the standard error of the mean. For A-C cells were grown in 96 well plates for 7 days at 28 °C. For D, settling rate was measured as an indicator of cell-to-cell attachment. B-C: Significant differences between treatments are indicated by brackets with asterisks, as determined by the Kruskal–Wallis test followed by pairwise Wilcoxon comparisons (p < 0.05). Treatments without brackets/asterisks were not significantly different. D: No significant differences were found according to one-way ANOVA and Tukey’s HSD test (*p* < 0.05).

Differences were observed among the strains examined based on the assessment of relative bacterial growth in vitro on the rich medium PD3. LA-Y3C_WT and its mutant LA-Y3C_1088 reached the highest OD_600_ compared to all other strains (Fig 3A). BB08_WT and its mutants had the lowest planktonic growth. Interestingly, the only significant difference between WT and mutants of the same strain was noted for LA-Y3C, whose WT showed significantly (*p* = 0.0002) lower planktonic growth than its mutant LA-Y3C_1088 (Fig 3B). As expected, trends for biofilm growth were the opposite to the ones observed for planktonic growth. Biofilm formation was highest for BB08–1_WT and its mutants (Fig 3C), and lowest for AlmaEm3_WT and mutants (Fig 3C). LA-Y3C_WT formed (*p* = 0.0002) significantly higher biofilm formation than its mutant LA-Y3C_1088 (Fig 3C), whereas the biofilm formation of AlmaEm3_1088 was significantly higher than AlmaEm3_WT (*p* = 0.0376). For cell settling rates, which is a measurement of cell-to-cell attachment, we found no significant differences when comparing WT with its mutants in all three tested strains (Fig 3D). The lower setting rate (less cell-to-cell attachment) between BB08–1_2741 and its WT and LA-Y3C_1088 and its WT was not significant (Fig 3D).

### Deletion of Locus_1088 in strain LA-Y3C increased bacterial movement in vitro

**Fig 4:**
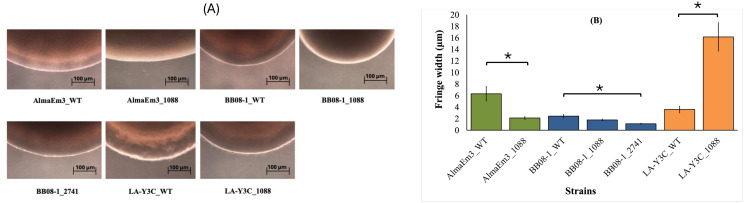
Twitching motility assay for wild type and mutant strains. (A) Pictures showing the motility fringe surrounding bacterial colonies spotted on PD3 plates for 5 days. Scale bar = 100 µm. (B) Measurement of colony fringe width for both wild type and mutant strains. Significant differences between treatments are indicated by brackets with asterisks, as determined by the Kruskal–Wallis test followed by pairwise Wilcoxon comparisons (p < 0.05). Treatments without brackets/asterisks were not significantly different. Vertical lines represent the standard error of the mean.

The mean fringe width of different colonies was calculated as a measurement of twitching motility. The fringe width of LA-Y3C_1088 (mean average of 16 μm) was significantly (*p* < 0.0001) higher (~ + 450%) than LA-Y3C_WT (mean average of 3.5 μm). On the other hand, a significant (*p* = 0.0015) but slightly lower (~ −65%) fringe was observed for mutant AlmaEm3_1088 (mean average of 2.12 μm) as compared to its AlmaEm3_WT (mean average of 6.3 μm). Similarly, both BB08−1 mutants showed less movement than their WT counterpart, with a significant (*p* < 0.0001) but a small reduction (~ −52%) observed for BB08–1_2741 (mean average of 1.15 μm) compared to BB08–1_WT (2.43 μm) (Fig 4B).

### Symptom development and plant colonization in blueberry plants

**Fig 5:**
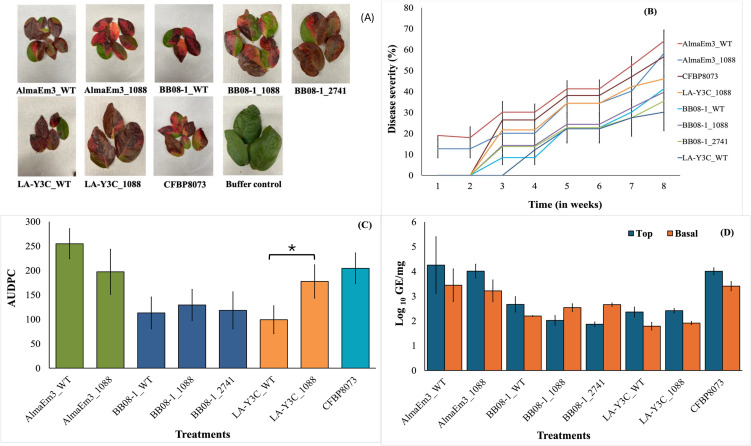
Symptoms caused by wild type and mutant *X. fastidiosa* strains on blueberry plants. Symptoms on blueberry plants were observed over a period of eight weeks after the first symptoms appeared on any plant, ~ 15 weeks post inoculation. (A) Representative pictures of leaves from plants infected with *Xf* or with a buffer control. (B) Disease progress curve of strains over time. (C) AUDPC of wild type and mutant strains tested in greenhouse experiment on blueberry. (D) Bacterial population determined by qPCR. Statistical differences were determined by Kruskal-Wallis test followed by Pairwise Wilcox test (*p* < 0.05). In C, brackets with asterisks indicate significant differences among treatments. In D, no brackets with asterisks are shown as no significant differences were found among treatments. Vertical lines represent the standard error of the mean.

In greenhouse studies, blueberry plants inoculated with all the mutants and WT strains studied here exhibited typical leaf scorch symptoms (Fig 5A). However, the severity of symptoms varied among strains. Infection with AlmaEm3 caused more symptoms than all other strains, but no significant differences were observed between AlmaEm3_WT and its mutant AlmaEm3_1088 (Fig 5B, 5C). BB08–1_WT and its mutants caused similar levels of disease severity, and no significant differences were observed among their AUDPC values (Fig 5B, 5C). Curiously, LA-Y3C_1088 produced significantly more severe symptoms than LA-Y3C_WT (*p* = 0.0257) (Fig 3C). In addition, symptoms caused by LA-Y3C_1088 were noticeable about two weeks earlier than in LA-Y3C_WT (Fig 5B). The *Xf* subsp. *fastidiosa* strain CFBP8073 originally from coffee, caused severe symptoms not significantly different from the most severe strain tested here AlmaEm3 (Fig 5). We also assessed bacterial populations at two locations in each plant, at the basal level close to the infection point, and the top part, to determine how the strains colonized the xylem system and moved inside the plant. No significant differences were observed when comparing leaves in the two positions for the same strain, nor in comparisons of WT vs. mutants at each position (top or basal) (Fig 5D).

## Discussion

Here we describe the functional characterization of genomic loci that are uniquely-encoded in *Xf* subsp. *multiplex* strains that cause disease in blueberry. The deletion of Locus_1088 in strain LA-Y3C increased virulence in blueberry and movement in vitro, while reducing biofilm formation. This locus corresponds to a non-annotated CDS within blueberry-infecting *Xf* genomes available on NCBI that hypothetically encodes a protein of unknown function. Deletion of this locus also caused a frameshift mutation in a RelE/ParE toxin-encoding gene (Fig 1) likely leading to a non-functional CDS. Additionally, the genomic neighborhood of Locus_1088 includes a type II toxin-antitoxin (TA) system composed of a ParD antitoxin and another copy of a RelE/ParE antitoxin directly downstream to Locus_1088 (Fig 2). In general, the phenotypes of mutants in other strains were either non-significantly or slightly different than their respective WT, therefore we are focusing on LAY-3C_1088 for this discussion.

Type II TA systems are comprised of a pair of genes encoded in the same operon in which one gene encodes a stable toxin that disrupts specific cellular processes, and the other encodes a labile antitoxin that inhibits the toxin [43]. They are diverse and widespread in bacterial genomes, regulating various cellular processes. They can function as stress response modules or plasmid addiction systems, and influence phenotypes such as biofilm formation, motility, persistence, and virulence [44–46]. Toxins in the RelE/ParE toxin superfamily display different biological roles. RelE type toxins act as mRNA endoribonucleases [47], while ParE type toxins inhibit DNA gyrase [48]. A search using the NCBI Conserved Domains tool (www.ncbi.nlm.nih.gov/Structure/cdd/wrpsb.cgi) revealed that the toxin gene uniquely encoded by blueberry-infecting *Xf* strains (adjacent to Locus_1088 and frameshifted in this study) is an mRNA-degrading endonuclease, being thus a RelE type toxin. In *E. coli*, RelE functions as a global inhibitor of translation activated during amino acid starvation [49]. Due to the mRNA-degrading action of many toxins of TA systems, they act as global regulators of transcriptome profiles. Another example is the MqsR toxin of the MqsRA system that regulates formation of persister cells and biofilm in *E. coli* [43,50]. In *Xf*, the MqsRA system regulates growth and formation of persister cells in response to copper stress, biofilm formation, motility, and virulence in sweet orange plants [46,51] by acting as a global regulator [52]. The RelE/DinJ TA system has been shown to specifically impact in vitro planktonic growth and biofilm formation of *Xf* [51], and its virulence and proliferation in grapevines [53]. These studies and our results collectively suggest how TA systems affect important phenotypes of *Xf* that impact virulence in host plants. A major difference between these TA systems previously studied in *Xf* is that they are found in the majority of *Xf* genomes available at NCBI, while the one studied here (adjacent to Locus_1088) is restricted to just a few blueberry-infecting strains. However, aspects that still need to be elucidated include: 1) the specific function of the toxin adjacent to Locus_1088; 2) the impact of having an orphan toxin without the cognate antitoxin, which has not been previously described in any organism to the best of our knowledge; 3) whether the ParD antitoxin of the adjacent TA system can counteract the action of this sole toxin; and 4) if the observed phenotypes in strain LA-Y3C are due to deletion of Locus_1088, the frame shift mutation in the toxin gene or both; and 5) the mechanism by which the identified locus and/or toxin affect host specificity.

From the two loci selected after a comparative genomics analysis [6], Locus_1088 was the only one causing a pronounced change in the phenotypes assessed, although it was significant only in two of the three strains tested. The phenotypes of LA-Y3C_1088 of increased planktonic growth, less biofilm and more movement suggest that the mutant could be more successful than the WT at colonizing the xylem network, but less effective at being transmitted by insect vectors [2]. There are some discrepancies about the role of biofilms in disease development by *Xf*, with some research suggesting more biofilm formation can be an indication of more symptoms, while other research indicates the contrary [12]. We hypothesize that in our study the more severe and earlier developing symptoms observed with LA-Y3C_1088 could be a result of planktonic cells widespread in the xylem and more efficiently interacting with the host and possibly triggering excessive defense responses that appear as symptoms [54]. Therefore, the role of Locus_1088 in blueberry specificity may rely on helping the pathogen to remain less motile in biofilms, therefore colonizing less of the xylem network, triggering fewer plant immune responses, and reducing virulence. More in-depth research is needed to test this hypothesis. We speculate that Locus_1088 may be a negative regulator of spread and colonization of *Xf* in its host plants. Unfortunately, we could not detect significant differences in bacterial population spread from the point of inoculation in blueberry. The lack of differences can be attributed to the use of multiple infections per plant over time, a single time point when bacterial populations being measured, and the fact that we used potted plants in the greenhouse, which have a much smaller size than can be found in the field. More research is needed to determine if Locus_1088 is important for colonization only in blueberry or in other hosts as well.

Interestingly, when we searched for the two loci (Locus_1088 and Locus_2741) selected as putatively associated with blueberry infection against *Xf* genomes, only a handful of strains isolated from non-blueberry hosts contained both loci. One of those strains was *Xf* subsp. *fastidiosa* CFBP8073, which was intercepted in France in coffee plants originating from Mexico [25]. Our greenhouse assays showed that this strain is as virulent as the control strain AlmaEm3 isolated from blueberry. Symptoms and bacterial populations in the host were similar between these two strains. Bacterial leaf scorch in blueberry has not been detected in Europe yet, but previous studies showed that a few *Xf* strains isolated from Europe can cause symptoms in blueberry [10]. The results shown here underscore the dangers of importing infected plants to new locations and illustrate the possibility of predicting host specificity based on a few loci. This has the potential to be useful since it is very cumbersome and time-consuming to assess host specificity among *Xf* strains [12]. Nevertheless, further experimentation with a higher number of strains is needed to determine if these loci are predictive of blueberry infection.

## Conclusions

In this study, we identified a locus hypothetically coding for a protein with unknown function and containing the first two nucleotides of a type II toxin that is involved in virulence in a strain isolated from blueberry. The lack of phenotypic reproducibility among the strains tested here suggests that the regulation of this locus is variable and unknown, but further studies are needed to elucidate this. Moreover, the identification of a blueberry-infecting strain, CFBP8073, based on genome content opens the possibility of further studies to develop a way to predict host specificity that will be useful in areas where infections are recent, therefore determining which nearby plant hosts are more in danger of infection by the introduced strains.

## Supporting information

S1 Table Metadata. Metadata of all strains used to generate the pan genome on which gene presence/absence correlations were estimated.
(XLSX)

S2 Table Pan Genome Info. Pan genome list, annotation, and table of gene presence or absence in each strain used for the analysis. (XLSX)

S3 Table Scoary *Vaccinium spp*. Genes whose presence/absence were correlated with the host genus *Vaccinium spp.* (XLSX)

S4 Table Primers used in this study. (DOCX)

S5 Table Nucleotide and amino acid sequences, annotation, size of loci (in bp) and locus tags of annotated gene in identified loci used for knockout. (DOCX)
