# Supplementary material for: A genomic locus uniquely encoded by blueberry-infecting Xylella fastidiosa strains affects motility and biofilm formation in vitro, and virulence in planta
Source: PLoS One. 2026 Apr 3;21(4):e0346230. doi: 10.1371/journal.pone.0346230 (PMC13048404; doi:10.1371/journal.pone.0346230)
Supplement: S4 Table — (DOCX) [file pone.0346230.s004.docx]

## **Table S4 Primers used in this study.**

| **Primer name** | **Sequence (5’-3’)** | **Source** |
| --- | --- | --- |
| **Primers used for obtaining knockout constructs for Locus_1088^a^** | | |
| 1088_UP_F | GAAAGGACAGGTTTAAGAC | This study |
| 1088_UP_R | GTCAGCAACACCTTCTTCACGAGGCAGACGCCCCTTGAGCCTCGTCACAGAGAT | This study |
| 1088_Dn_F | CATCAGAGATTTTGAGACACAACGTGGCGAGTGGTAGCGATGTACCAATA | This study |
| 1088_Dn_R | CACATCCTTTGGAGTGACACC | This study |
| 1088_BB08_UP_F | GGAAAGATAGCAGAAAAAATC | This study |
| 1088_BB08_UP_R | GTCAGCAACACCTTCTTCACGAGGCAGACCCGACACGCTCAGTGGATAGTG | This study |
| 1088_BB08_Dn_F | CATCAGAGATTTTGAGACACAACGTGGCGCCCCTTGAGCCTCGTCACAGAGAT | This study |
| 1088_BB08_Dn_R | GGTGTGCCCACTGATGCGATAG | This study |
| **Primers used to confirm deletion of Locus_1088^b^** | | |
| 1088_F_conf | GCAAAAGGGCATCGATCACG | This study |
| 1088_R_conf | GCACAGTATCGAGAATGAAGTTTC | This study |
| 1088_Up_F_conf | CACTCTTGCCATATTGAATG | This study |
| 1088_BB08_F_conf | CGTTTCTGAGAATGATGC | This study |
| 1088_BB08_R_conf | CCTTAGCAAAAGGGCATCGATCACG | This study |
| 1088_BB08_UP_F_conf | GCAACACCTATGGCTGGAAATG | This study |
| **Primers used for obtaining knockout constructs for Locus_2741^a^** | | |
| 2741_UP_F | GCCACTCATACCGACTCATTAAAC | This study |
| 2741_UP_R | GTCAGCAACACCTTCTTCACGAGGCAGACAGCAGGATAGGGATAAGC | This study |
| 2741_Dn_F | CATCAGAGATTTTGAGACACAACGTGGCTACTACACTCCTACCGTGCCCTATC | This study |
| 2741_Dn_R | CTTCCGCAACACACTCGGCAAC | This study |
| **Primers used to confirm deletion of Locus_2741^b^** | | |
| 2741_F_conf | GTGGAGCGGGCAAAGAAACAC | This study |
| 2741_R_conf | GAATCTTTATCTGGTGCTTCTG | This study |
| 2741_UP_F_conf | CGTACACCGATACGCGATG | This study |
| 2741_AlmaEm3_Dn_R_conf | CGTTAGCTCAGTCGGTAGAG | This study |
| **Pair of primers used to amplify the Km resistance cassette from pUC4K^c^** | | |
| Kan_F | GTCTGCCTCGTGAAG | Kandel et al., 2018 |
| Kan_R | AAGCCACGTTGTGT | Kandel et al., 2018 |

^a^Primers used for obtaining the knockout constructs for Locus_1088 and Locus_2741. The upstream and downstream sequences of AlmaEm3_1088 and LA-Y3C_1088 shared 99.38% and 100% identity, respectively, enabling the use of a single primer pair for the amplification of both targets and different primers in case of BB08-1. ^b^ Deletion of loci were confirmed using primers designed from nucleotide sequence of loci and confirmation of upstream and downstream regions. ^c^Km resistance cassette was amplified from pUC4K by using Km forward and reverse primers from the previous study.
