# Supplementary material for: A genomic locus uniquely encoded by blueberry-infecting Xylella fastidiosa strains affects motility and biofilm formation in vitro, and virulence in planta
Source: PLoS One. 2026 Apr 3;21(4):e0346230. doi: 10.1371/journal.pone.0346230 (PMC13048404; doi:10.1371/journal.pone.0346230)
Supplement: S5 Table — (DOCX) [file pone.0346230.s005.docx]

## **Table S5 Nucleotide and amino acid sequences, annotation, size of loci (in bp) and locus tags of annotated gene in identified loci used for knockout.**

| **Locus** | **Nucleotide sequence^a^** | **Amino acid sequence^a^** | **Annotation^b^** | **Size in bp** | **Locus tag of annotated gene in the identified loci (strain)^c^** |
| --- | --- | --- | --- | --- | --- |
| **Locus_1088** | ATGTCCTTAGCAAAAGGGCATCGATCACGGGAAGCAGGTCGTCTTCCAACGTGGCGCGATGCTGCCCCTTGGCAGGGATTGGTGTCTTGTGGCGCATCATTCTCAGAAACGTTCGTTTGTGACAAGCACGCCAAGACAACCGGCATGACTTGTGGAATCAATGCGTTGCAATTCTCGAAAATAGTTCTTGAATCTTTATTCTCGATACAATGCTATTACAACGAGTTTCGATAAGAGTGGTAGCGATGTACCAATA | MSLAKGHRSREAGRLPTWRDAAPWQGLVSCGASFSETFVCDKHAKTTGMTCGINALQFSKIVLESLFSIQCYYNEFR-EW-RCTN | (Few base pairs) Type II toxin-antitoxin system mRNA interferase toxin, RelE/StbE family + Non-annotated CDS | 256 bp | LZ759_RS11330 (LA-Y3C)  KBP49_RS11475 (AlmaEm3)  KBP48_RS11845 (BB08-1) |
| **Locus_2741** | ATGTTGAATAATCAAGAATCTTTATCTGGTGCTTCTGACTTGGGTGTTCTCAAACAGCATTCGCGTATTGTTGATTTTGATGTTGAAGAGGTGCGCACTTTAATTTCTGATATTCAGGATATTAGATCCAAACTAATTTCATCTTTTAAAATTTTAGATAAGTCTTCTTCACTTGTTGATGAAGTGGGCATTCATGATGCCAAAGAAGCGCTAAAAACTAGTATTAGTAAGATTCTTTATGCTGATGACGAATTACTATATCTTGCTGGGCTTTTGTCTGAGAGTGTTTCTTTGCCCGCTCCGCTTTTCTGA | MLNNQESLSGASDLGVLKQHSRIVDFDVEEVRTLISDIQDIRTK LISSFNILDKSSSLVHEVGNHDAKEALKISISKILYADDELLHLAGLLSESVSLPAPL F | Hypothetical protein | 312 bp | KBP48_RS02745 (BB08-1)  KBP49_RS02795 (AlmaEm3) |

^a^For Locus_1088, nucleotide and amino acid sequence are provided for the deleted region including Type II toxin-antitoxin system mRNA interferase toxin and the non-translated region. For Locus_2741, nucleotide and amino acid sequence for hypothetical protein are shown. ^b^Annotations and locus tags are shown for identified loci for all three strains available on NCBI.
